# Noise measurements as a proxy to evaluating the response to recommendations in times of crisis: An update analysis of the transition to the second wave of the CoViD-19 pandemic in Central Stockholm, Swedena)

**DOI:** 10.1121/10.0003778

**Published:** 2021-03-17

**Authors:** Romain Rumpler, Siddharth Venkataraman, Peter Göransson

**Affiliations:** The Marcus Wallenberg Laboratory for Sound and Vibration Research (MWL), Department of Engineering Mechanics, KTH Royal Institute of Technology, SE-100 44, Stockholm, Sweden

## Abstract

Sweden stands out among the other European countries by the degree of restrictive measures taken towards handling the 2019 coronavirus outbreak, associated with the CoViD-19 pandemic. While several governments have imposed a nationwide total or partial lockdown to slow down the spread of the virus, the Swedish government has opted for a recommendation-based approach together with a few imposed restrictions. In a previous contribution by the authors, the impact of the Swedish strategy was observed through the monitored variation of the city noise levels during a period associated with the so-called “first wave” of the pandemic in Stockholm. A very strong impact of these recommendations was shown on the evolution of the noise levels in central Stockholm. This highlighted the potential of acoustic sensor networks both for enforcement of regulation and monitoring of the effectiveness of their implementation. The present contribution presents a follow-up to this urban noise monitoring in central Stockholm, Sweden, for the period leading to the so-called “second wave” of the pandemic in Europe. Both the evolution of adherence to the recommendations and the impact of the recurrence of cases combined with reinforced recommendations are observed through the evolution of the measured noise levels. While the measurements show a gradual lower level of compliance, in particular, past the summer break, these also show again a rapid response to the reinforced recommendations issued by the authorities in mid-fall of 2020. These observations thus confirm the potential associated with detailed urban noise monitoring, for instance here acting as a proxy to evaluating the response to recommendations or restrictions in times of crisis.

## I. INTRODUCTION

Following the global spread of the 2019 novel coronavirus, SARS-CoV-2, causing the contraction of CoViD-19 by millions of people around the world, the authors presented a study shedding some light on the response to the lenient mitigation strategy adopted by the Swedish authorities during the initial spread of the virus in Stockholm, Sweden.^1^ With the analysis of noise level measurements in a strategic central location of the city center, the study highlighted both the impact of the propagation of the virus on human activities and the prompt reduction of these activities upon the issuing of recommendations and restrictions by authorities, including transport-related noise emissions. It also reported, however, that after a few weeks of strong reduction in the measured noise levels, where the average levels were in the range of the two quietest public holidays of dominant importance in the Swedish culture, a steady increase in these levels was subsequently observed until the end of June 2020, which marked the end of the analysed data. This suggested a gradual return to ordinary levels of activity, although this point was never reached in the time-frame of the study. The present contribution consists of a follow-up to this early analysis, thus providing further insight into the response to the recommendation-based approach, on a period extending to the so-called second wave of propagation of the virus, which led to an updated set of recommendations and restrictions in Sweden.

This unprecedented situation has given rise to a range of studies analysing both the diversity of responses to the crisis and its impact on our societies and environments. Recent studies have thus been focusing on these different policy responses. Desson *et al.*^2^ have, for instance, focussed on the early response and associated impact on the outbreak of the pandemic for Germany, Austria, and Switzerland, in comparison to most of their European neighbour countries such as Belgium, France, or Italy. Sweden, with its recommendation-based approach completed by a few restrictions, has also been in focus for this rather unique response,^3^ and was included in a comparison of four representative divergent responses together with China, France, and Japan.^4^ The distinct trajectories of these four responses were highlighted to be contextually dependent, influenced by the response of the respective populations, the distinct institutional arrangements, and diverse national cultural orientations. Taking advantage of the numerous cases of strict lockdowns inducing severely reduced human activities, numerous studies have evaluated the associated reduction in environmental footprint,^5–9^ even highlighting a more contrasted assessment when considering factors beyond polluting emissions.^10^ However, since the authors' initial contribution in the special context associated with the Swedish mitigation strategy, relatively few acoustic studies have been reported in connection with a reduction of urban noise pollution. Among these, Asensio *et al.*^11^ reported a substantial reduction in noise levels, in the range from 4 to 6 dB(A) in the day, evening, and night averaged levels taken separately, during the lockdown in the city of Madrid, Spain, extended from March to June 2020. Although these reductions exceed the ones observed in the center of Stockholm^1^ in the same period, the authors observe that a significant part of this reduction is associated with a change in the intraday patterns of these levels, which was only marginal in Stockholm. Covering a similar period, associated with the so-called “first wave” of the pandemic in Europe, a measurement campaign over 12 monitoring stations in the city of Dublin, Ireland, also reported a significant reduction in hourly averaged equivalent levels for all stations during the lockdown period.^12^ Highlighting the importance of city-wide sound monitoring networks in order to assess the effectiveness of noise pollution mitigation strategies, this contribution echoed the authors' claim of the relevance of such networks in order to follow and adapt a dynamic set of recommendations and restrictions in times of crisis.^1^ This interest in city-wide noise monitoring networks is further highlighted in studies associated with noise mapping evaluations.^13,14^ The follow-up analysis presented in the following, covering the period leading to the so-called “second wave” of the CoViD-19 pandemic in Stockholm, provides yet another argument in this direction.

## II. EVOLUTION OF NOISE LEVELS PAST THE FIRST WAVE AT A STRATEGIC MONITORING STATION

### A. Summary of the methodology: Noise level fluctuations with respect to long-time averages

Following the previous contribution focussed on the outbreak of the pandemic,^1^ the same strategic location was selected for the analysis of the monitored noise levels due to the significant contribution from multiple and relevant sources of noise associated with urban activity, at the corner of a busy crossroad in central Stockholm. The different types of acoustic measures, such as *LA_eq_*, *LA*_90_, *etc.*, are calculated in 1-min intervals and presented in a relative sense to reference average levels since the interest is in their fluctuation with respect to pre-pandemic noise levels and activity. The calculation of these reference averages is summarized in the steps below. Let *T* be the set associated with all the time instances *t*, i.e., here minutes, part of a time period of interest. These instances *t* are uniquely referred to by their associated date *t_date_* (i.e., day, month, and year), and time *t_hm_* (i.e., hour-minute timestamp of the form hh:mm), such that

$$t=(t_{\mathrm{date}},t_{hm}).$$ (1)

In order to allow for a distinction of reference noise levels by different days (i.e., according to a specific day of the week), such sets as the following are introduced:

$$t_{\mathrm{day}}\in \{t_{\mathrm{Mon}},t_{\mathrm{Tue}},\ldots ,t_{\mathrm{Sun}}\},$$ (2)

where, for instance, *t_Mon_* refers to the set {Monday}. This presumes the introduction of a function getday, such that

getday(t)∈{Monday,Tuesday,Wednesday,Thursday,Friday,Saturday,Sunday}. (3)

Note that the definition of a day is in practice adapted from the conventional 00:00–23:59 time period in order to match the urban noise level patterns observed.^1^

On the basis of the notations adopted in Eqs. (1)–(3), a reference time period $T_{\mathrm{Ref},hm}^{\mathrm{day}}$, associated with a given set *t_day_*, and for each intraday timestamp hh:mm indexed by the subscript $\cdot_{hm}$, may be defined as

$$T_{\mathrm{Ref},hm}^{\mathrm{day}}=\{t|t\in T\wedge \mathrm{getday}(t)\in t_{\mathrm{day}}\wedge t_{hm}=\text{hh}:\text{mm}\},$$ (4)

subsequently leading to the associated average reference noise level ${\tilde{L}}_{\mathrm{Ref},hm}^{\mathrm{day}}$,

$${\tilde{L}}_{\mathrm{Ref},hm}^{\mathrm{day}}=\frac{\sum_{t\in T_{\mathrm{Ref},hm}^{\mathrm{day}}}^{}L(t)}{\mathrm{card}(T_{\mathrm{Ref},hm}^{\mathrm{day}})},$$ (5)

where *L*(*t*) corresponds to the acoustic noise measure associated with time instance *t*, in dB, and $\mathrm{card}(T_{\mathrm{Ref},hm}^{\mathrm{day}})$ to the cardinality of the time period subset. Note that pre-determined periods, from June 1, 2019 to July 31, 2019, and from December 16, 2019 to January 14, 2020, are excluded from the complete time period *T* used to calculate the reference time period, in Eq. (4), in order to avoid the influence of special periods such as summer and winter breaks, which tend to deviate from the normal conditions of interest here.

A date-wise noise level difference, for a given *t_date_*, may be defined as the average of the level difference between the minute-wise noise levels and the reference level ${\tilde{L}}_{\mathrm{Ref},hm}^{\mathrm{day}}$ associated with the suitable time, such that $\mathrm{getday}(t)\in t_{\mathrm{day}}$. This requires first to define the set of minute-wise noise levels difference associated with a given day *t_date_*, such that

$$\Delta L(t_{\mathrm{date}})=\{L(t)-{\tilde{L}}_{\mathrm{Ref},hm}^{\mathrm{day}}|\mathrm{getday}(t)\in t_{\mathrm{day}}\wedge t_{hm}=\text{hh}:\text{mm}\},$$ (6)

leading to the average noise difference for *t_date_*, given by

$$\tilde{\Delta L}(t_{\mathrm{date}})=\frac{\sum_{x\in \Delta L(t_{\mathrm{date}})}^{}x}{\mathrm{card}(\Delta L(t_{\mathrm{date}}))}.$$ (7)

The average difference for a particular day is thus calculated according to Eq. (7) with a reference average value established according to Eq. (5). In practice, a distinction is made between two different day-groupings across the week:^1^ (i) Weekdays (i.e., from Sunday 18:00 to Friday 17:59), and (ii) Weekend days (from Friday 18:00 to Sunday 17:59).

### B. Noise measurements and analysis

#### 1. Measurement of noise level fluctuations

**FIG. 1.**
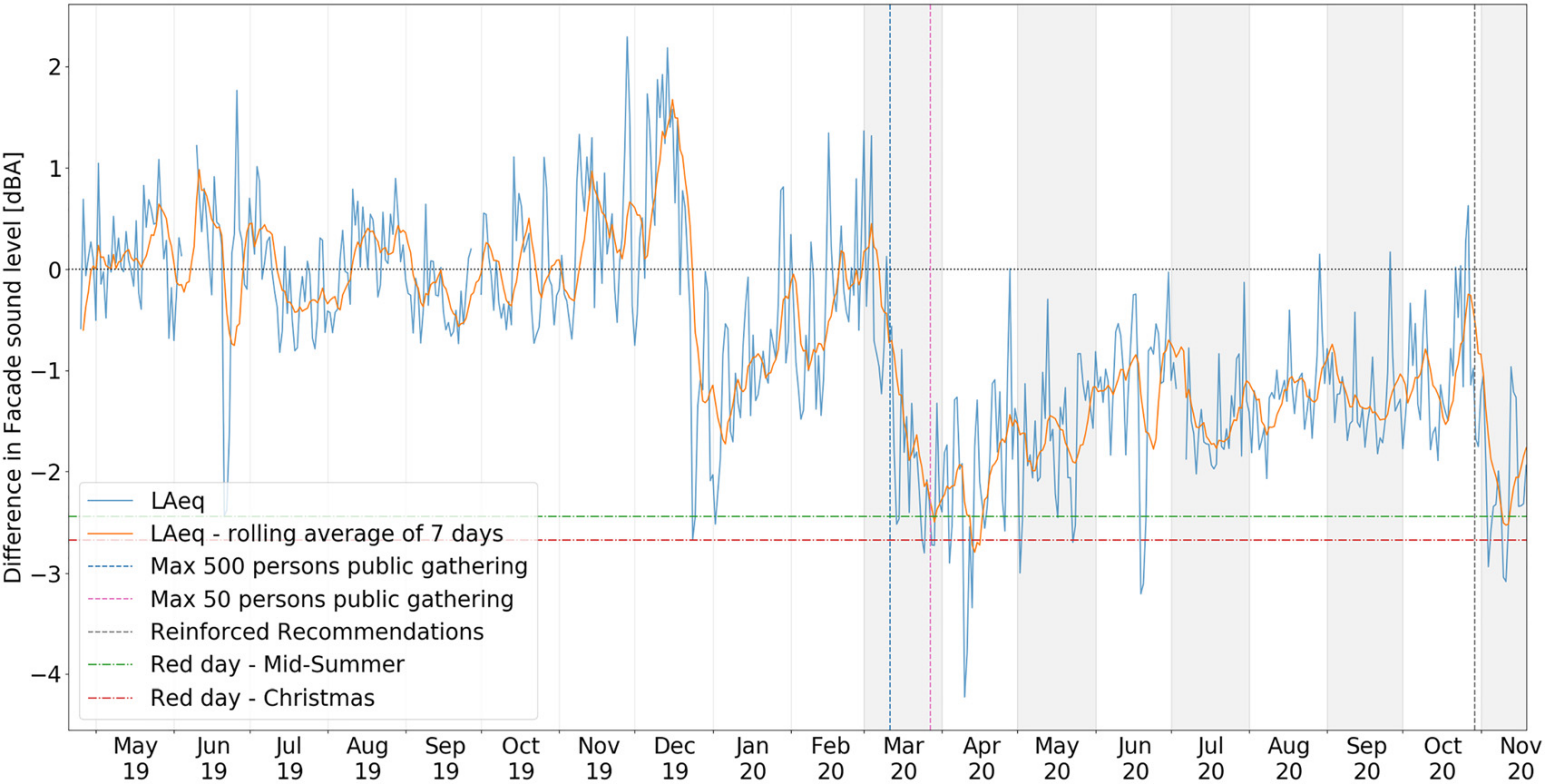
(Color online) Overview of the noise level fluctuation for the period April 2019 – November 2020, shown with 1-day resolution and 7-day rolling average. Horizontal dashed lines highlight levels associated with major red days in 2019. The vertical dashed line highlights major recommendations issued by the Swedish authorities.

**FIG. 2.**
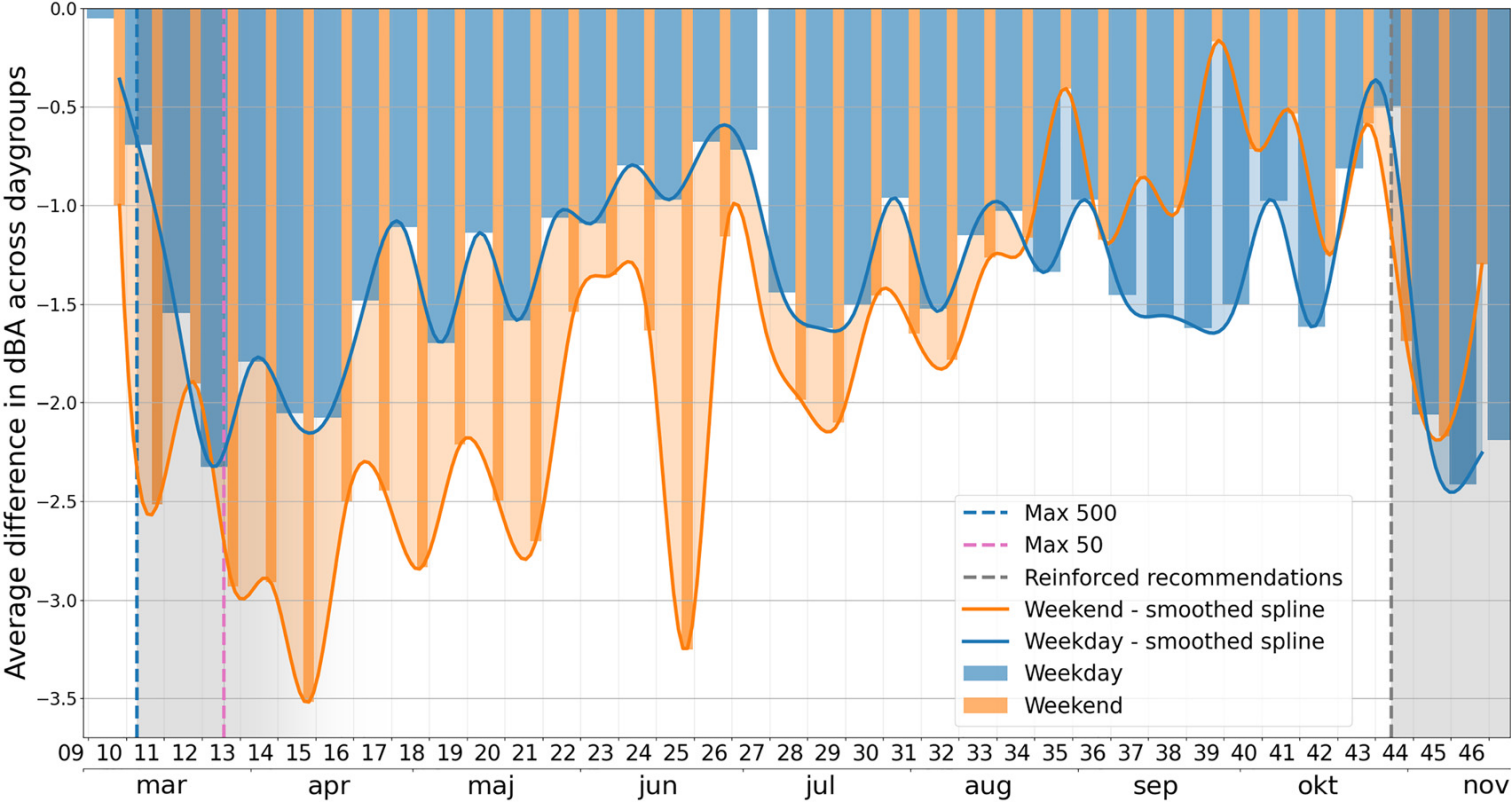
(Color online) Noise level fluctuation with respect to the long-time reference average, taking into account groupings according to weekday and weekend patterns. The gray areas highlight the periods of major recommendations issued at the start of the first and second “waves” of major propagation of the virus.

The measurements of noise levels were conducted in direct extension of the initial contribution by the authors:^1^ a noise level monitoring device installed with the microphone on the façade of a building located at the corner of a busy crossroad in central Stockholm. The location was specifically chosen due to the significant contribution from multiple sources of noise associated with urban activity, such that it may be considered as representative of human-activity in central Stockholm, including traffic from public transport, individuals and freight traffic, entertainment (cinemas, bars, cafés, restaurants and nightclubs in the area), local residential life, etc. The noise levels were logged continuously at this location from the middle of April 2019, which provides a good background basis for the evaluation of the impact associated with the reduced activity due to the emergence of CoViD-19 and the measures taken by the authorities. Similar to the initial contribution, the evolution of the averaged noise levels is presented in terms of fluctuations with respect to the 2019 reference levels. An overview of these noise level fluctuations for the entire period from April 2019, to the last week of November 2020, the date of the latest available data, is presented in Fig. 1. Together with daily fluctuations of measured noise levels with respect to the averaged reference from 2019, levels reached on the two major Swedish public holidays in 2019 are highlighted with horizontal dashed lines, and major recommendations issued by the authorities are marked with vertical dashed lines. The interested reader is referred to the original contribution^1^ for further details on the period up to July 2020, for which a summary of the main conclusions is provided in Sec. II B 2. The period extending beyond this is further detailed in Fig. 2, as well as further analysed in Sec. II B 3. In Fig. 2, an important distinction is made between weekdays and weekend days, assuming that the former are primarily related to economic activities during working hours and that the latter reflect behaviours associated with the general population. The data presented ranges from March 2020, i.e., slightly before the start of the first wave of the CoViD-19 pandemic in Stockholm, to until the last week of November 2020, the latest available data.

#### 2. Main observations during the “first wave” of widespread propagation

The fluctuations associated with the first wave, until the end of June 2020, were previously analyzed, with the following main conclusions:^1^

• A sharp drop of the weekend levels on the first weekend of March 2020, initiated slightly before the first recommendations issued by the Public Health Agency of Sweden (“Folkhälsomyndigheten,” or FoHM) the following week;
• A sustained reduction of both weekday and weekend levels over a period of about one month from these recommendations and restrictions (the period where a series of recommendations and restrictions were issued by FoHM is highlighted in grey in Fig. 2);
• A gradual but steady increase of the average levels from the middle of April 2020;
• Over the entire period leading to the summer break (end of June), a higher drop of the averaged noise levels associated with the weekend days than those associated with the weekday levels, suggesting that self-imposed restrictions following the recommendations by authorities may have been observed in good agreement with the ones taken for economic activities.

The summer period, extending from the end of June 2020 to approximately the middle of August 2020, is considered to be of lesser interest in the present follow-up, given the complexity associated with the summer activities: part of the urban population leaving the city, potentially partly compensated by an increase in activity of those observing their holidays in the city. It is nevertheless noteworthy that the overall drop, weekdays and weekends considered, seems to have been relatively stable over this period, in a range between –1 dB(A) and –2 dB(A).

#### 3. A clear shift leading to updated recommendations for the “second wave”

The period that follows immediately the summer holidays, from the middle of August 2020, is however of key interest. The drop of the averaged noise levels from that point on to the middle of October 2020, week 42, appears to be in continuation of the summer activity with, however, a major difference: for the first time since the start of the pandemic, the drop of the average noise levels associated with the weekend days is consistently lower than that associated with the weekdays. This is highlighted in Fig. 2 by the color-filling between the smoothing splines, turning to blue over the period from week 35 to week 43. This may be a reflection of the continued compliance to the recommendations and restrictions associated with the economic life (e.g., remote work when possible), contrasting with a potential gradual adoption of a casual attitude towards the situation by a part of the population tending to neglect some of the self-imposed reduction in activity observed before the summer. The sharp reduction in the number of cases reported during the summer may possibly be another underlying reason. This led to the lowest drop in levels observed over two consecutive weeks since the start of the pandemic, on week 43 and the first part of week 44.

On October 29, a Thursday, week 44, in the face of an increasing number of confirmed cases of people tested positive for CoViD-19, a trend which had started earlier in several other European countries, FoHM released a new recommendation for three regions in Sweden, including Stockholm.^15^ This included, among others, a recommendation to refrain from visiting indoor environments such as shops, malls, museums, libraries, concert halls, or sports arenas. It went further by recommending, if possible, to avoid having physical contact outside of one's personal household, thus dissuading any arrangement or participation in social gatherings. This was the strongest recommendation issued since the start of the pandemic, initially intended to be maintained until November 19. Figure 2 highlights the immediate response observed through a sudden reduction of the averaged noise levels. Note that this sharp reduction of the averaged noise levels in response to this reinforced recommendation is also very clearly visible in Fig. 1, where the associated date is marked with a vertical dashed line. In Fig. 2, starting with the weekend immediately following this recommendation and the subsequent week, the reduction was comparable to the one observed following the initial set of recommendations in the middle of March. Though the drop during weekend averages remained lower than those associated with this initial set of recommendations, it is noteworthy that for the first time since the summer, the average noise level drop of weekends shortly exceeded those associated with the preceding weekdays.

## III. CONCLUSION

In this short communication, a follow-up analysis of the fluctuation of measured noise levels at a busy crossing in central Stockholm compared to 2019 levels, confirms the potential of acoustic monitoring in the context of Smart Cities, as a source of information about urban life, e.g., in connection with regulations, the degree of compliance, and associated impact.

In the present case, in the context of the recommendation-based mitigation strategy in response to the propagation of the novel coronavirus (2019-nCoV) in Sweden, the noise level measurements, leading to the “second wave” of widespread propagation, have highlighted a clear shift compared to the situation prior to the summer break: the impact of the pandemic on the average weekend levels became consistently inferior to the impact on the week levels. While the latter remained in the range of the noise level reduction observed prior to the summer, i.e., approximately [−1,−1.6] dB(A) during weeks 35–42, the weekend average drop rarely went beyond –1 dB(A) in that same period. This may highlight a form of weariness by a part of the population, not reflected to the same degree during the weekdays where the implementation of the recommendations in the economic activities may have been mostly maintained over this period.

Interestingly, the lowest combined drop observed for week and weekend average levels was observed on week 44 and the preceding weekend. Toward the end of that same week, the strongest recommendations since the start of the pandemic were issued, leading to an immediate response whose dynamic is clearly captured by the average noise level reduction on the order of level drops observed following the first few recommendations in March 2020. These observations confirm both the immediate impact of the recommendations and restrictions implemented by the Swedish authorities, as well as the potential associated with detailed urban noise monitoring, for instance, here acting as a proxy to evaluating the response to recommendations or restrictions in times of crisis.
